# Contrasting Weather and Stocking Effects on *Eucalyptus* Initial Coppice Response in Brazil

**DOI:** 10.3390/plants13223254

**Published:** 2024-11-20

**Authors:** Pietro Gragnolati Fernandes, Clayton Alcarde Alvares, Túlio Barroso Queiroz, Pedro Vitor Pimenta, Jarbas Silva Borges, James Stahl, Flávio Teixeira Mendes, Amanda Souza, Gustavo Matheus Silva, Gualter Guenther Costa da Silva, Sara Bezerra Bandeira Milhomem, Rosilvam Ramos de Sousa, Rodrigo Eiji Hakamada

**Affiliations:** 1 Department of Forest Science, Federal Rural University of Pernambuco, Recife 52171-900, PE, Brazilrodrigohakamada@usp.br (R.E.H.); 2 Suzano S.A. Company, Itapetininga 18207-780, SP, Brazil; 3 Department of Forestry Sciences, São Paulo State University (UNESP), Botucatu 01049-010, SP, Brazil; 4 Sylvamo of Brazil, Mogi Guacu 13845-901, SP, Brazil; 5 Dexco S.A., Buri 18290-000, SP, Brazil; 6 Klabin S.A., Telemaco Borba 84260-000, PR, Brazil; jstahl@klabin.com.br; 7 Aço Verde do Brasil, Acailandia 65930-000, MA, Brazil; flavio.mendes@ferroeste.com.br; 8 Gerdau Aços, Tres Marias 39205-000, MG, Brazil; amanda.souza5@gerdau.com.br; 9 Vallourec South America, Paraopeba 35774-000, MG, Brazil; 10 Department of Forest Science, Federal University of Rio Grande do Norte, Natal 59078-970, RN, Brazil; gualtermve@gmail.com; 11 Department of Forest Sciences, University of São Paulo, Piracicaba 13418-900, SP, Brazil

**Keywords:** silviculture, initial growth, productivity, climate gradient

## Abstract

In *Eucalyptus* plantations, coppice rotations often yield less than initial rotations. The TECHS project (Tolerance of *Eucalyptus* Clones to Hydric, Thermal and Biotic Stresses) studied short rotation coppicing across a 3000 km gradient. The main objective of this work was to compare the survival, sprouting, and initial growth of *Eucalyptus* clones managed and to examine factors that might influence the productivity of the coppice rotation: climate, genotypes, and stocking. Eight of the TECHS sites spread from latitudes 6° S to 30° S were included in the coppice study, with 17 genotypes at each site. The initial rotation had been planted at a 3 m × 3 m spacing and also in a spacing trial at densities from 500 to 3500 trees ha^−1^. Six months after harvesting the initial *Eucalyptus* rotation, average survival was 88%, with tropical clones showing over twice the sprouting biomass (6.7 vs. 2.9 Mg ha^−1^) and four times the woody biomass compared to subtropical clones (4.7 vs. 1.1 Mg ha^−1^). Greater initial water deficits had stronger sprouting and growth. Clones with higher belowground carbon allocation in the initial rotation performed better in coppicing, and precipitation became more influential after 12 months. Drought and spacing trials significantly affected growth.

## 1. Introduction

Coppicing, one of the oldest silvicultural systems in the world, involves regenerating new trees from surviving stumps [1,2,3]. This practice offers significant cost savings compared to establishing new forests, as it reduces expenses related to site preparation, planting, and weed control [4,5]. Coppicing not only provides economic benefits but also enhances the resilience of forest ecosystems by maintaining genetic diversity and promoting faster recovery after disturbances, making it a sustainable option for forest management.

Sprouting ability varies significantly among species [6], as well as provenances and genotypes within species like *Eucalyptus* [7]. Genetic factors, including the number of epicormic buds and lignotubers, the development of the root system, and susceptibility to fungal infection, can influence the species’ ability to sprout [8,9]. Environmental factors also influence sprouting responses to silvicultural treatments. Typically, yields decline across coppice rotations, with variations influenced by climate conditions, where higher rainfall may lead to higher yields in coppice rotations compared to initial rotations [10]. Few studies have tracked individual growth across rotations, with some reporting no correlation between growth in different rotations, even across regions with different tropical climates [11].

Stocking levels, in conjunction with site and genotype characteristics, can influence root system development, subsequently impacting wood growth and survival [12]. While many studies have explored stocking effects in short rotation planted forests, research on this aspect in coppice rotation is limited, with few exceptions, such as a study with willow stands [13]. The response to changes in stocking may differ in coppice rotations due to the preexisting root system, potentially resulting in faster regeneration post-harvest [14].

To gain comprehensive insights into why growth patterns differ between coppice and planted rotations, a wide range of experimental treatments and site factors must be considered. Our investigation is focused on a coppice rotation at eight of the original 35 sites of the TECHS project—Tolerance of *Eucalyptus* Clones to Hydric, Thermal and Biotic Stresses [15]. The second phase of this project, known as the PCoppice project (Cooperative Program on Sprouting Productivity of *Eucalyptus* Clones, https://www.ipef.br/pcoppice/ (accessed on 6 October 2022)), specifically investigates the influence of climate and stocking levels on sprouting and productivity in coppice rotation of the most commonly planted *Eucalyptus* clonal genotypes in Brazil.

Therefore, as part of the PCoppice project, this paper aims to address the following 5 questions:

(1) Do tropical clones exhibit greater sprouting biomass and wood biomass compared to subtropical clones?
(2) Are the most productive clones in the initial rotation also the most productive at the beginning of the coppice rotation?
(3) Do dominant trees from the initial rotation maintain dominance in the coppice rotation?
(4) What is the impact of climate on sprouting and initial growth of genotypes in coppice rotation?
(5) How does the interaction between climate and stocking affect the growth of clones at the beginning of the coppice rotation?

By exploring these questions, this study seeks to provide valuable insights into the factors influencing the early stages of coppice growth, ultimately contributing to more effective management strategies for *Eucalyptus* plantations in varying climatic conditions.

## 2. Results

**Figure 1:**
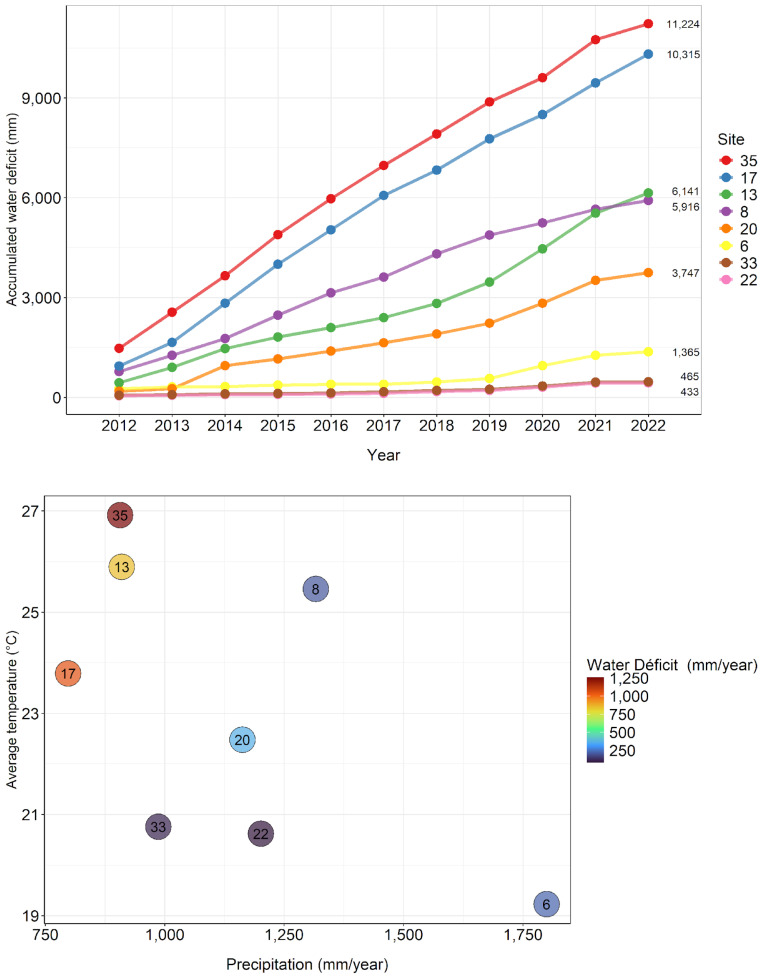
Accumulated water deficit from 2012 to 2022 (**above**), precipitation, average temperature, and water deficit in the first year of the coppice rotation at the PCoppice sites (**below**).

The experimental site with the lowest accumulated water deficit since planting in 2012 was site 22, with 433 mm, while the largest deficit occurred at site 35, with 1224 mm. Sites 35, 17, and 13 are the hottest and driest in the coppice rotation. Site 8 had a higher water deficit in the initial rotation, but in the coppice, rotation is wetter so far (Figure 1).

The average sprouting rate across all sites and all genotypes measured at 6 months was 88%. Of the 17 clones tested, 15 showed a sprouting rate above 80%. Clones O6 and L5 had lower sprouting rates, 63.5 and 29.3%, respectively.

**Figure 2:**
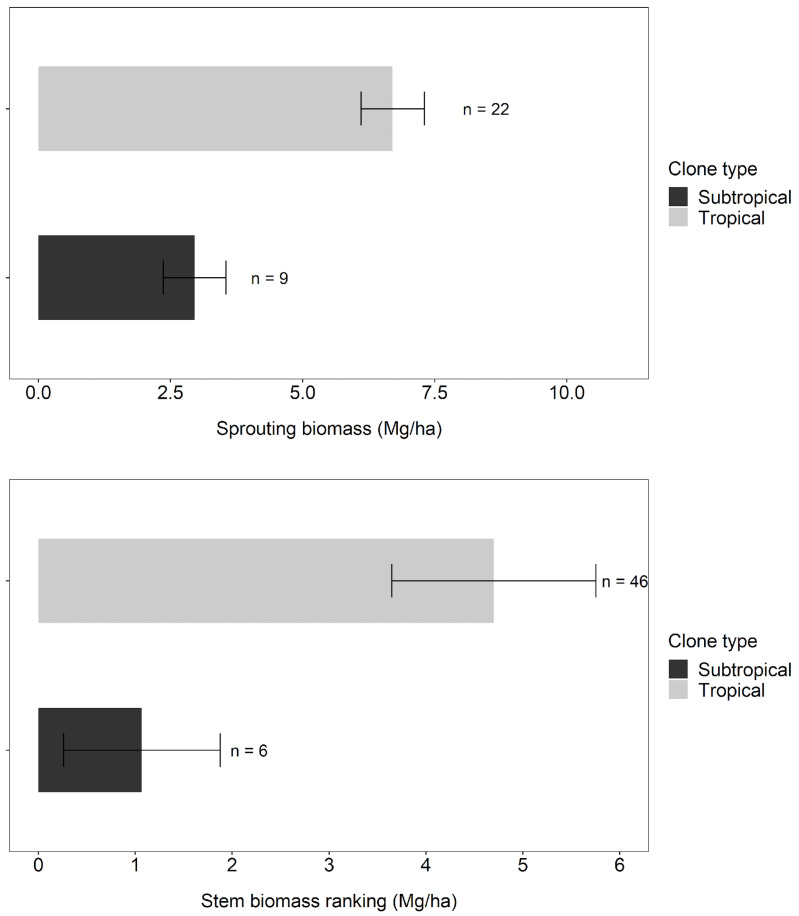
Sprouting biomass averages at 6 months (**above**) and wood biomass averages at 12 months (**below**) of clones classified as tropical and subtropical, where n represents the number of plots of each type. The error bar indicates the standard error of the mean.

Tropical clones, especially those with *E. urophylla* in their genetics, had higher sprouting and woody biomass than subtropical clones. Sprout biomass at 6 months for tropical clones (6.7 Mg ha^−1^) was more than double that of subtropical clones (2.9 Mg ha^−1^, Figure 2). The difference between these classes of clones increased 4-fold at 12 months (4.7 Mg ha^−1^ for tropical, 1.1 Mg ha^−1^ for subtropical, Figure 2).

**Figure 3:**
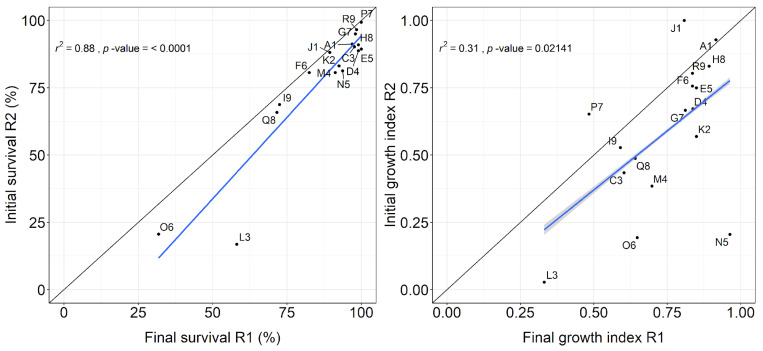
Relationships between final survival in initial rotation and initial survival in coppice rotation grouped by clone (**left**), final growth index in initial rotation, and initial growth index in coppice rotation (**right**). The black line represents the 1:1 ratio, and the blue line represents the linear regression.

Clones with high survival and growth rates in initial rotation also tended to have high rates at the beginning of coppice rotation. This relationship is stronger for survival than for growth (Figure 3).

Some clones exhibited considerable variations in growth between the initial and coppice rotations. Clone P7 and J1 exhibited a notable increase in growth, with an increase of 35% and 23%, respectively. In contrast, clones N5 and O6 exhibited a significant decrease in growth rate, with a reduction of approximately 75% and 70%, respectively (Figure 3).

**Figure 4:**
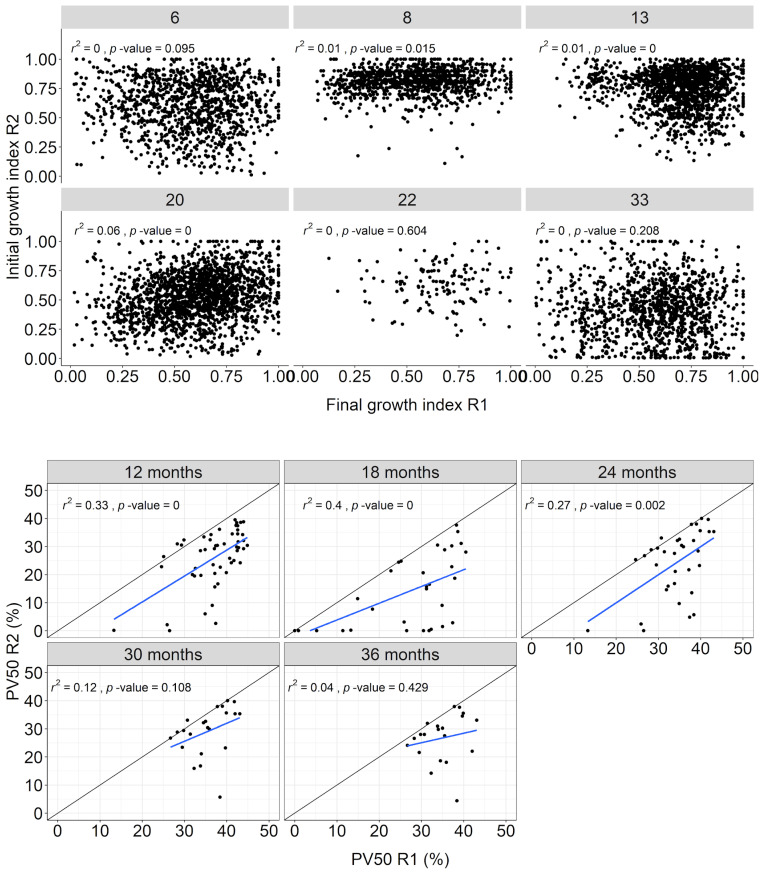
Relationships between final growth index in initial rotation and initial growth index in coppice rotation at individual level by site (**above**), final uniformity (PV50) in initial rotation, and initial uniformity in coppice rotation by age (**below**). The black line represents the 1:1 ratio, and the blue line represents the linear regression.

Growth of individual trees in the initial rotation was generally not a clear predictor of growth in the coppice rotation. Therefore, dominant trees in the initial rotation can become dominant in the coppice rotation, indicating that factors that led to the relative success of individual trees in the initial rotation generally did not carry over into the coppice rotation (Figure 4, above). Changing growth at the individual level affects plot uniformity in coppice rotation. The initial similarity in uniformity (PV50) between the two rotations decreased with advancing age in the coppice rotation. In the first 2 years of the coppice rotation, within-plot uniformity correlated moderately with uniformity in the initial rotation (r^2^ between 0.27 and 0.4), but the correlation declined substantially in the next year. The plots that showed the highest uniformity in the initial rotation tended to show lower uniformity in the coppice rotation (Figure 4, below).

High stress from limited water and high temperature in initial rotation seemed to increase height at 6 months and wood biomass at 12 months in coppice rotation. At 6 months, the water stress of the coppice rotation does not seem to affect sprouting growth. However, at 12 months, the stem biomass starts to obtain a weak positive correlation with the rainfall of coppice rotation (0.29) and zero with the water deficit (0.14). Correlations with the average temperature in the coppice rotation remain high, indicating that warmer and rainier sites are performing better in the early growth phase of coppice rotation (Table 1).

**Figure 5:**
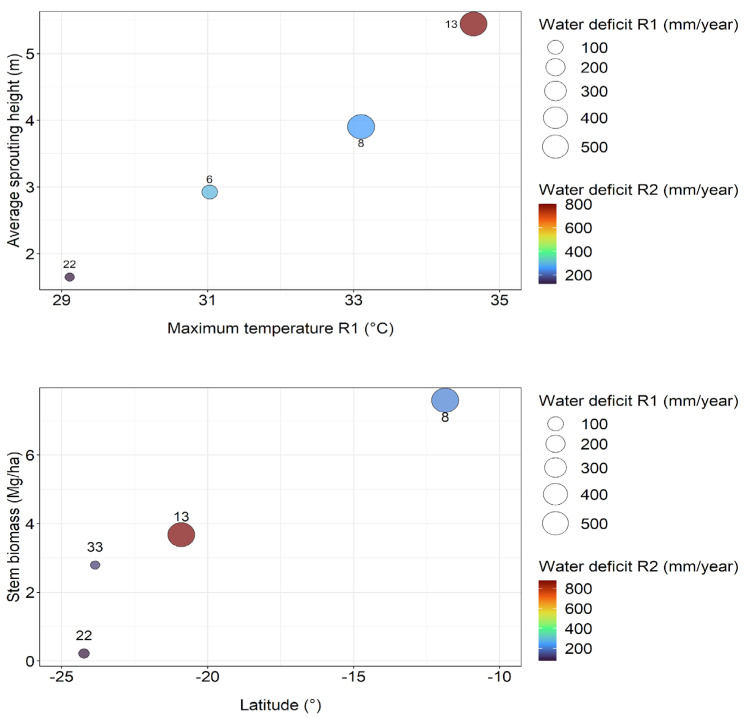
Relationships between sprouting height at 6 months (**above**) stem biomass at 12 months (**below**), and climatic variables show that the driest sites in the initial rotation became the most productive at the beginning of the coppice rotation.

The initial rotation of the TECHS Project showed that cooler and moister sites had greater yields at the end of the rotation than drier and warmer sites, but the early growth of the plantations was indeed greater on the water-stressed sites. The coppice rotation showed the same pattern in the first three years. Sites with the highest water deficit in initial rotation were the most productive at the beginning of coppice rotation. However, as age advances, water deficit in coppice rotation can reduce site productivity (Figure 5).

**Figure 6:**
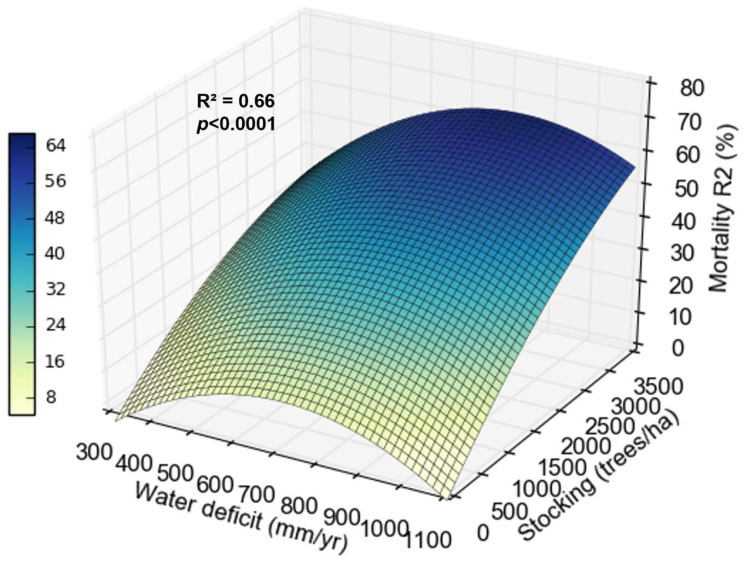
Relationship between mortality in the second rotation (R2), water deficit, and stocking.

Mortality in coppice rotation was influenced by stocking and water deficit. On sites with 300 mm year^−1^ water deficit, mortality was 0 to 30% between 500 and 3500 trees ha^−1^. On sites with 1000 mm year^−1^, the mortality went from 0 to 70% between 500 and 3500 trees ha^−1^ (Figure 6).

**Figure 7:**
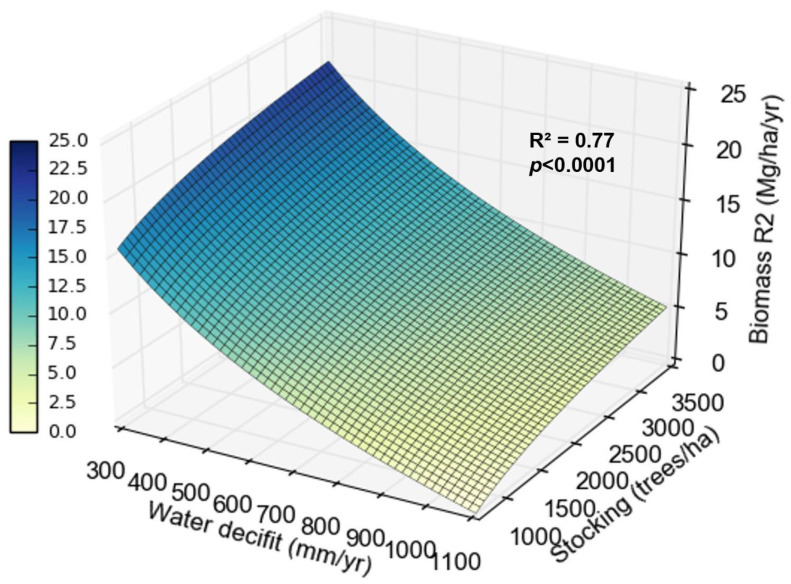
Relationship between biomass production in the second rotation (R2), water deficit, and stocking.

Biomass production at the beginning of the coppice rotation was also influenced by water deficit and stocking. On sites with lower water deficit (300 mm yr^−1^), biomass increased by about 7 Mg ha^−1^ yr^−1^ between widest and tightest stockings. On sites with greater water deficit (1000 mm yr^−1^), the biomass increased less, only about 3 Mg ha^−1^ yr^−1^ between the widest and tightest stockings (Figure 7).

## 3. Discussion

### 3.1. Do Tropical Clones Exhibit Greater Sprouting Biomass and Wood Biomass Compared to Subtropical Clones?

Tropical clones produced more sprout biomass and faster initial growth, especially those with *E. urophylla* in their genetics (Figure 2). We speculate that tropical clones allocated more C3 belowground in the initial rotation, supporting better coppice growth. Campoe et al. [16] documented that the carbon allocation to the aboveground and belowground parts of 5 TECHS clones at the end of the initial rotation followed patterns consistent with the region where they were developed. According to the author, the higher the water stress in the environment, the higher the proportion of belowground biomass. The P7 clone, for example, developed in regions with high temperatures and water deficit, allocates more carbon to the roots than subtropical clones developed in regions of low temperatures and high rainfall.

Species with high regrowth capacity tend to have around five times more carbohydrates in the roots than those that do not regrow [17,18]. As a consequence of this strategy, these species tend to produce less biomass in the aboveground part and present lower growth rates. This general trend across species and sites was similar to the pattern found in clone P7. This clone was the least productive in the initial rotation, with an average MAI of 21 m^3^ ha^−1^ year^−1^. Its low productivity was a reflection of its greater carbon allocation to the root system [16]. Perhaps clone P7 stumps and root systems had larger stores of carbohydrates to invest in new sprouts compared to other clones with lower belowground C allocation from the initial rotation. This speculation would warrant direct measurements of carbohydrate stores belowground (across clones and sites), as well as the duration of these stores in supporting sprouts that are increasing their own photosynthesis over time.

### 3.2. Will the Most Productive Clones in the Initial Rotation Be the Most Productive at the Beginning of the Coppice Rotation?

Overall, the results showed a tendency for the most productive clones in the initial rotation to be the most productive in the early growth stage of the coppice rotation (Figure 3). Amâncio et al. [19] obtained similar results when comparing several clones of *E. grandis*, *E. urophylla* × *E. grandis*, *E. saligna*, and *E. urophylla* in first and coppice rotation in a breeding program. About ¾ of the best clones in their initial rotation continued to be best in the coppice rotation. The relationship between survival in the first and the coppice rotation was very strong because, naturally, survival at the beginning of the coppice rotation is directly related to the number of surviving strains at the end of the initial rotation.

The growth index reflects the ranking and distances between the yields of the clones at each site, and it showed a moderate correlation between the first and second rotations (Figure 3, right). This is because some clones showed large variations in their positioning between the end of the initial rotation and the beginning of the coppice rotation. Two clear examples are clones P7 and N5, which may represent the extremes of the effect of carbon allocation to the root in initial rotation on the potential for sprouting and initial growth in coppice rotation. Clone P7 was developed in a tropical region with high temperatures and dry summer (As), and N5 was developed in a subtropical region with high rainfall and low temperature (Cfb). Another four subtropical clones follow N5 in the group of clones that lost more than 30% growth rate between the two rotations: L3, O6, M4, and K2.

We found some stability in the ranking of the clones between the two rotations, these early rankings may not persist to the end of the rotation. The ranking of clones in the initial rotation shifted over time [20], so results from the coppice rotation may still change.

### 3.3. Will the Dominant Trees in the Initial Rotation Remain Dominant in the Coppice Rotation?

The dominance status of individual trees and plot uniformity did not generally continue into the coppice rotation (Figure 4), in line with results from Pereira Filho et al. [11]. Natural or operational factors, such as shading or damage to the stumps at harvest time, can alter dominance relationships at the individual level. A stronger relationship between root and shoot growth in smaller trees, as opposed to dominant ones, could also account for the shifting hierarchy observed among trees. By applying the equation developed by Ledo et al. [21] to our dataset, we discovered that trees that are 20% smaller would have a root-to-shoot ratio of 25%, in comparison to the 21% found in trees that are 20% larger. This altered relationship would lead to a greater accumulation of carbohydrates below ground, ultimately resulting in similar growth between dominant and dominant trees.

### 3.4. How Does Climate Affect Sprouting and Initial Growth of Genotypes in Coppice Rotation?

The tropical sites, which suffered the most water and heat stresses during the initial rotation, had better initial sprouting and growth in the coppice rotation (Figure 5).

Throughout the growth of the coppice rotation, water deficit can reduce the biomass production of the clones. At 6 months, site 13 was the most productive, with a high value of water deficit in initial rotation (562 mm year^−1^) and in coppice rotation (762 mm year^−1^). At 12 months, site 13 lost the position of the most productive site to site 8, which also had a high water deficit in the initial rotation (570 mm year^−1^) but not in the coppice rotation (265 mm year^−1^). The fact that at 6 months, site 13 was the most productive site, and at 12 months, it lost this position to site 8 indicates that throughout the growth of the coppice rotation will depend in large part on current rainfall rather than on water stress in the prior rotation. On the other hand, site 33 was one of the most productive in the initial rotation, showing the lowest water deficit (34 mm year^−1^), and even with a low water deficit in the coppice rotation (76 mm year^−1^) showed a lower average wood biomass than the sites with higher water stress (Figure 5). It will be very interesting to see if the whole-rotation yields of the coppice rotation indicate higher growth in cooler, moister sites (as in the initial rotation) or the warmer, drier sites (as in the first half of the coppice rotation).

The justifications for the faster initial growth of the tropical sites may be related to the survival strategies of the genotypes in more stressful environments. During the initial rotation, in sites with greater water deficit, the trees allocated more carbon to the roots than in sites with greater rainfall and milder temperatures [16]. Whittock et al. [22] found that individuals of *E. globulus* developed less lignotuber in more humid environments, indicating that water stress stimulates the development of this structure. So, it is important to investigate the root biomass of the clones at the PCoppice sites to confirm the hypothesis that drier sites in the initial rotation are more productive at the beginning of the second rotation due to the higher amount of carbon in the root system.

### 3.5. How Does the Interaction Between Climate and Stocking Affect the Growth of Clones at the Beginning of the Coppice Rotation?

Mortality increased at the beginning of the coppice rotation with increasing stocking and water deficit (Figure 6). The effect of stocking on water relations is well studied in *Eucalyptus* in the initial rotation but scarce in the coppice rotation. Because the root system is already established, the influence of the water deficit might be weaker. Sites with the highest water deficit showed lower productivity gains between wider and tighter stockings, indicating that in these locations, increasing stocking does not reflect a significant increase in biomass production per hectare. The yield difference between the tighter and wider stockings dropped by 50% for every 200 mm more water deficit (Figure 7). This pattern may result from a greater reduction in water availability per tree with tighter stocking, increasing mortality, and reducing the increase in productivity. In sites with high water availability, the increase in stocking is reflected in significant increases in biomass. Binkley et al. [15] and Hakamada et al. (in. prep) also observed that subtropical sites (with lower water deficits) showed greater productivity responses to stocking than tropical sites in the initial rotation.

Bernardo et al. [23] analyzed the effect of stocking on the growth and distribution of biomass in individuals of *E. camaldulensis*, *E. pellita*, and *E. urophylla*. The authors concluded that, in addition to affecting growth, stocking had an effect on biomass partitioning between above and below ground. At wider stockings (833 trees ha^−1^), *E. camaldulensis* and *E. urophylla* showed increases of around 10% in the proportion of root biomass compared to tighter stockings (2222 trees ha^−1^). So, in the wider stockings, the trees are larger, and the biomass partitioning to the roots is greater, resulting in root systems with higher amounts of carbon. The authors [23] end their paper by concluding that “since larger root systems may increase future coppice growth yields, the effect of differences in allocation to the root system on future productivity needs to be evaluated”. Considering the positive effect of the increased amount of carbon in the root system on sprouting vigor, the results of this work may provide insights into the hypothesis raised by [23]. The lower amount of carbon in roots at the tighter stockings may be the explanation for the high mortality rates at sites with high water deficit, making it necessary to plant 3000 more trees ha^−1^ at these sites to achieve a productivity increase of only 3 Mg ha^−1^. Therefore, although the trees begin the coppice rotation with a developed root system, it is not sufficient to guarantee the survival of very tight stands in sites with high water deficits.

## 4. Materials and Methods

### 4.1. Site Description

**Figure 8:**
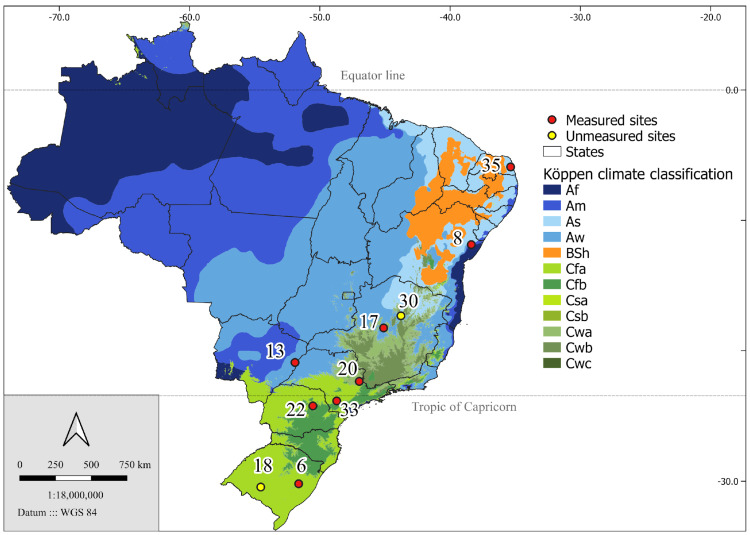
Location of the 10 PCoppice project sites and the climatic types in which they are located, according to Köppen’s climatic classification. The two sites in yellow were not considered in this work. Description of the climatic types of the Koppen classification: Af (tropical rainforest climate), Am (tropical monsoon climate), As (tropical savanna climate with dry summer), Aw (tropical savanna climate with dry winter), BSh (hot semi-arid climate), Cfa (humid subtropical climate), Cfb (oceanic climate), Csa (hot-summer Mediterranean climate), Csb (warm-summer Mediterranean climate), Cwa (monsoon-influenced humid subtropical climate with dry winter), Cwb (subtropical highland climate with dry winter), Cwc (cold subtropical highland climate with dry winter).

**Figure 9:**
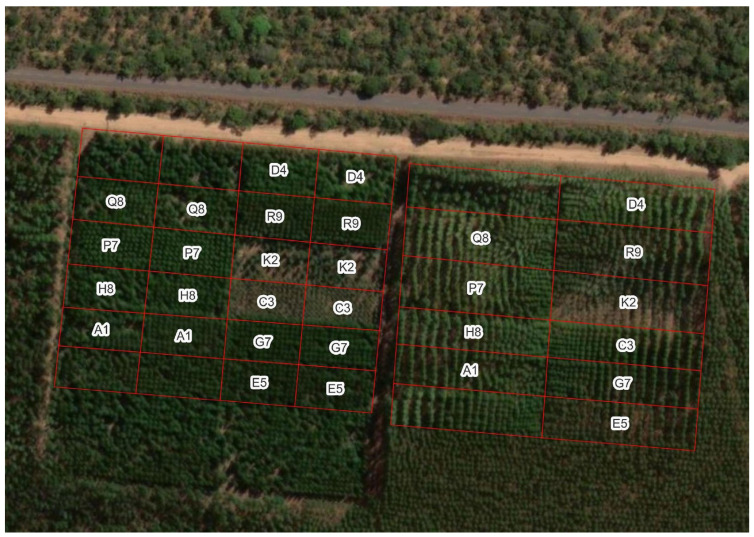
Clonal test (**left**) and spacing test (**right**) at site 17, in Três Marias—Minas Gerais. Plots without the clone acronym have genotypes not evaluated in PCoppice.

The eight experimental sites (Figure 8) are distributed between latitudes 6° S and 30° S (Table 2), with mean annual temperatures differing by up to 6.5 °C and mean annual precipitation with a range of 600 mm. Each experimental site comprised plots designed for testing clones with 120 trees, planted in 8 rows of 15 trees at the standard plantation spacing of 3 × 3 m (1100 trees ha^−1^) in tropical Brazilian sites. The first 5 trees in each row were used for destructive sampling in the initial rotation, so the useful plot consists of 80 trees (8 rows × 10 trees). Additionally, three of the sites continued a spacing trial with plots of 7 rows by 27 trees, with varying distances between trees and stand densities ranging from 450 to 14,000 trees ha^−1^ (Figure 9). For this study, we considered the trees planted at stand densities ranging from 500 to 3500 trees ha^−1^ as useful plots.

### 4.2. Genotypes

Seventeen commercial *Eucalyptus* clones were evaluated: nine tropical clones (A1, C3, D4, E5, G7, H8, P7, Q8, R9), and eight subtropical clones (F6, I9, J1, K2, L3, M4, N5, O6), according to its genotype and climate of the breeding pipeline site (Table 3). The genotypes were grouped by the breeders of the TECHS group, as shown in the paper published by Binkley et al. [15] (refer to Figure 2 on page 3). Genotypes previously classified as plastic in the TECHS study (A1, C3, K2, and Q8) were reclassified as tropical or subtropical in this study. This reclassification was necessary because they did not exhibit truly plastic behavior during the initial rotation.

### 4.3. Climatic Data and Water Balance

The climate data were sourced from the NASA/POWER platform—NASA Langley Research Center POWER Project, providing daily estimates of climate variables derived from predictive models utilizing images from the MERRA-2 satellite at a 0.5 × 0.625-degree resolution [25]. Daily scale series of maximum, minimum, and average temperature (°C), dew point temperature (°C), precipitation (mm), relative humidity (%), wind speed at 2 m height (m s^−1^), surface pressure (kPa), and incident radiation (MJ m^−2^ day^−1^) were collected from 1 January 2010 (2 years prior to the planting of the initial rotation) to 31 December 2022. Accumulated degree days were calculated [26] as:

(1) $${DD}_{Accumulated}=\sum_{i}^{n}\left[\left(T_{min}-T_{i}\right)+\frac{\left(T_{max}-T_{min}\right)}{2}\right],$$

where *DD_Accumulated_* = accumulated degree days (°C), *T*_*min*_ = daily minimum temperature (°C), *T*_*max*_ = maximum daily temperature (°C), *T*_*i*_ = lower basal temperature of the crop (10 °C) [27], *n* = number of days.

The soil water balance was calculated using an adapted version of the Thornthwaite and Mather (TM) method [28], proposed by Pereira et al. [29]. The water holding capacity of each site was taken from Binkley et al. [16] (Table 2). Reference evapotranspiration (ETo) was calculated using the Penman–Monteith (PM) method [30]. This calculation employed the using the PM function from the SPEI package [31] within the R 4.4.0 software [32]. The computation of Eto relied on data for minimum temperature, maximum temperature, dew point temperature, wind speed, latitude, incident radiation, relative humidity, atmospheric pressure, and altitude. The main results obtained from TM water balance method include estimates of soil water storage, surplus, and water deficit.

### 4.4. Establishment of the Coppice Rotation

The original rotation of the TECHS project was harvested between 2019 to 2021. Different companies employed various harvesting system, including long log systems (Feller Buncher + Skidder), short log systems (Harvester + Forwarder, or Feller Buncher + tracer claw + Forwarder), and semi-mechanized cutting (chainsaw). Despite the system used, all stumps were cut at a height of 10 to 15 cm to avoid damage.

Ant control baits were applied approximately 2 months before harvest, adhering to local company protocols. Harvested stems were removed from the sites within a month, along with residues that could potentially shade or impede the growth of the new sprouts. Fertilizer applications followed the patterns used by each company in their commercial coppice areas, typically consisting of 1950 kg ha^−1^ of limestone, 66 kg N ha^−1^, 65 kg P ha^−1^, and 120 kg K ha^−1^ on average. Weed control was performed approximately twice until canopy closure, employing glyphosate at a rate of 2.88 kg of active ingredient ha^−1^, applied manually with care to avoid contact with tree leaves. After 12 months, weed control with the same method was performed on average once a year, depending on the degree of weed infestation.

**Figure 10:**
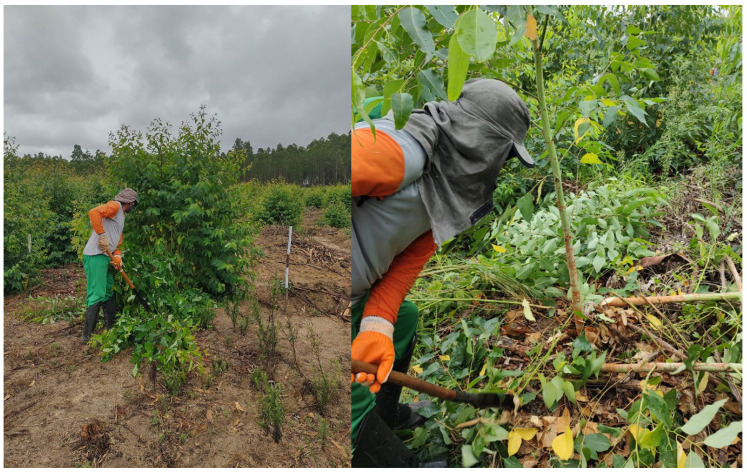
Stump before pruning (**left**) and pruned stump (**right**) at site 8, in Inhambupe—Bahia.

Multiple shoots per stump were pruned at 6 months post-harvest. However, pruning was delayed till about 12 months on sites 22 and 35 due to concerns regarding potential strong winds, allowing the sprouts to develop greater resistance. Across all sites, only the most vigorous sprout was retained, and in the event of mortality, two sprouts were maintained on the subsequent tree to uphold stand density (Figure 10).

### 4.5. Growth Measurements

The initial evaluation, conducted at 6 months pre-pruning, aimed to determine survival rates and estimate sprouting biomass for each genotype. Sprouting rate was defined as the percentage of stumps from the initial rotation that sprouted. Given that not all initially planted trees survived until the end of the initial rotation, survival was calculated based on the number of sprouted stumps relative to the original planting density. Sprout biomass estimation relied on allometries equations adjusted in this study, utilizing total height and the number of sprouts per stump. Allometric equations were developed with destructively sampled sprouts carried out to adjust the specific equations for each plot and general equations applicable to the genus *Eucalyptus*:

(2) y=1.41753h,

where y = shoot dry biomass in kg DM individual^−1^ and h = total individual height. R^2^ = 0.86.

To capture the variability in the height of individuals, four height classes were determined in each plot using the mean and standard deviation. Eight individuals per plot were sampled, with two individuals per height class. These selected individuals underwent uniform sprouting, where one or two main shoots were kept, and the rest were removed. The removed shoots were separated into leaves, twig, and wood, and each compartment had its wet weight measured in the field. Samples of each component were weighed and then dried in the laboratory. To factor in the biomass of the retained shoots on the stumps, an average dry biomass per removed shoot was calculated for each individual, and this average was added to the dry biomass of the removed shoots.

After pruning, forest inventories were conducted every six months until 24 months of age and annually thereafter. However, only site 20 was old enough to carry out annual measurements. Therefore, they were not used in comparative analyses between sites, only in the comparisons between growth in the initial rotation and in the coppice rotation on the same site. Height and DBH of the trees were measured using a Haglof clinometer and a diametric tape.

At sites that have not yet reached the expected age for destructive biomass sampling (36 months), dry mass (B, in kg tree^−1^) of wood was predicted using the linearized model of Schumacher and Hall [33], with a clone-specific intercept fitted by Mattos et al. [34]:

(3) $$lnBj=\beta 0j+1.8534\;ln\;ln\;\left(DBH\right)+1.1414\;ln\;ln\;\left(H\right)\;$$

where Bj is the dry wood mass in kg tree^−1^ of clone j, β0j is the specific intercept of clone j, DBH is the diameter at breast height in centimeters, and H is the total height of the tree in meters.

Due to the differences in age between the sites, a growth index was generated to represent the ranking of the clones in each site and allow for comparison between them even with different ages. The growth index was defined as a value that can range from 0 to 1, where 1 represents the productivity of the most productive plot of the site.

Thus, the indices of the other plots are defined by dividing their productivity by the productivity of the most productive plot on the site:

(4) $${GI}_{js}=\frac{P_{js}}{{Pmax}_{s}},$$

where ${GI}_{js}$ is the growth index of clone *j* at site *s*, $P_{js}$ is the productivity of clone *j* on site *s*, and ${Pmax}_{s}$ is the maximum productivity (yield of the most productive clone) of site *s*.

Thus, this index represents the position of a clone in relation to the most productive clone on the site, allowing comparison between clones regardless of the age of measurement.

To compare the uniformity between individuals in the plots, the PV50 index proposed by Hakamada [35] was used, which consists of the percentage of total biomass present in 50% of the smallest trees in the plot, including planting gaps. PV50 requires the ordering of trees from the smallest to the largest tree in individual volume and is calculated according to the following equation:

(5) $$PV50=\frac{\sum_{k=1}^{\frac{n}{2}}V_{ij}}{\sum_{k=1}^{n}V_{ij}},$$

where *PV50* = Accumulated percentage of the individual volume of the 50% smallest trees planted, *V* = individual volume of plot *i* at age *j* and *n* = number of trees planted in order (from smallest to largest).

### 4.6. Data Analysis

Comparisons between clones were made with descriptive graphs of sprout and woody biomass grouped by clone type. The averages were calculated using the available plots from each group, with a larger n for the group of tropical clones due to the greater presence of tropical sites in the experiment. The error bars indicate the standard error of the mean.

Growth differences from the initial rotation into the coppice rotation were compared at the individual level, at the plot level, and at the clone level, with graphical analysis and linear regression. The averages of the variables analyzed (survival, growth rate, and PV50) were calculated in the first measurement of the plots in the second rotation, and in the last measurement of the same plots in the initial rotation. For the results shown at the clone level, the averages of all the plots available for each genotype were calculated.

The relationships between climate variables and growth variables in the first and coppice rotation were examined with graphical analysis and Pearson correlation. The average sprouting height at 6 months and stem biomass at 12 months were calculated for the four sites with measurements at these ages, taking into account all the plots planted at each site. A correlation matrix was generated with the climatic variables, and the strongest correlations are shown in the results.

Finally, to verify the interaction between climate and stocking on the growth of the clones, three-dimensional plots were generated by Curve Expert to describe the behavior of mortality and biomass production at different stockings and water deficit ranges. All the other analyses were performed in R software [32].

## 5. Conclusions

The development of the root system in the initial rotation has a direct effect on the initial sprouting. Sites with high water stress promote a more pronounced development of the root system than humid sites. Furthermore, genotypes from drier regions tend to allocate proportionally more C to the roots.

This study presents the hypothesis that water stress conditions in the initial rotation may favor the sprouting of materials at the beginning of the coppice rotation. This is because sprout growth is mostly determined by the accumulation of reserves in the roots, especially in clones adapted to water stress conditions. However, after the first year of coppice rotation, rainfall is expected to have more of an effect on growth, favoring wetter sites.

We evaluated the survival and initial growth of the interaction of 17 genotypes, 8 contrasting climate sites, and densities between 500 and 3500 trees ha^−1^ in *Eucalyptus* clone sprouts. Despite the wide variation in factors, which makes the study unprecedented, we recognize some weaknesses in the methods. The existence of hybrid clonal materials from contrasting species, such as clone C3, a hybrid of *E. grandis* and *E. camaldulensis*, precludes the possibility of broadening the discussion about the response of a particular species since other clones with the same species can have completely different results. The absence of replications at the same site and the lack of standardization of treatments make it challenging to identify independent variables, as there are sites that do not have precisely the same genotypes. Nevertheless, this analysis with repetitions along the climatic gradient, in which the predictor variable becomes the site’s water deficit, has been employed in forestry and ecology studies and allows the population of interest to be broadened. Finally, it should be acknowledged that the evaluations were conducted at an early age, and the results may undergo significant changes over time. However, some studies conducted on fast-growing stands in Brazil have demonstrated that early growth results (up to one year) are highly correlated with the final outcomes of the study.

## Figures and Tables

**Table 1:** Pearson correlations between sprouting height at 6 months and stem biomass at 12 months with climatic variables of initial and coppice rotation.

Age (Months)	Variable	Initial Rotation			Coppice Rotation		
		Rainfall	Average Temperature	Water Deficit	Rainfall	Average Temperature	Water Deficit
6	Sprouting height (m)	−0.80	0.78	0.89	−0.48	0.89	0.81
12	Stem biomass (Mg ha^−1^)	−0.87	0.81	0.77	0.29	0.75	0.14

**Table 2:** The locations of the PCoppice sites spanned a gradient of 3000 km, 6.5 °C in mean annual temperature, and 600 mm of annual rainfall.

Site	Lat (Degrees)	Long (Degrees)	Altitude (m)	Average Annual ppt (mm) ^1^	Average Temperature (°C) ^1^	Soil Type	Water Holding Capacity (mm) ^2^	Average Annual Water Deficit (mm) ^1^
6	−30.19	−51.62	150	1576.0	20.0	Ultisol	145	124
8	−11.86	−38.37	218	1131.1	25.6	Ultisol	89	538
13	−20.9	−51.9	361	1062.3	25.2	Oxisol	87	558
17	−18.25	−45.1	806	914.3	24.1	Oxisol	76	938
20	−22.35	−46.97	633	1199.0	22.2	Oxisol	165	341
22	−24.23	−50.53	888	1458.5	20.8	Latosol	214	39
33	−23.85	−48.7	695	1386.7	20.6	Latosol	196	42
35	−5.90	−35.35	650	1047.4	26.5	Oxisol	89	1020

^1^ Annual averages considering the period from 2012 (initial rotation planting year) to the end of 2022. ^2^ Values obtained from [15].

**Table 3:** Genotypes evaluated in PCoppice ^1^.

Code	Genotype	Group ^2^	Climate of Origin ^3^
A1	*E. urophylla*	Tropical	Cwa
C3	*E. grandis × E. camaldulensis*	Tropical	As
D4	*E. grandis × E. urophylla*	Tropical	Aw
E5	*E. urophylla*	Tropical	Cwa
F6	*E. benthamii*	Subtropical	Cfb
G7	*E. urophylla*	Tropical	Cwa
H8	*E. grandis × E. urophylla*	Tropical	Am
I9	*E. dunnii*	Subtropical	Cfb
J1	*E. benthamii*	Subtropical	Cfb
K2	*E. saligna*	Subtropical	Cfb
L3	*E. urophylla × E. globulus*	Subtropical	Cfb
M4	*E. dunnii*	Subtropical	Cfb
N5	*E. dunnii*	Subtropical	Cfb
O6	*E. grandis*	Subtropical	Cfb
P7	*E. urophylla × E. brassiana*	Tropical	As
Q8	*E. grandis × E. urophylla*	Tropical	Af
R9	*E. urophylla*	Tropical	Aw

^1^ Table taken from Binkley et al. [15]. ^2^ Grouping by TECHS breeders [15]. ^3^ Köppen climatic classification [24].

## Data Availability

Data are contained within the article.
